# Correction: The lived experience of rejection sensitivity in ADHD - A qualitative exploration

**DOI:** 10.1371/journal.pone.0345112

**Published:** 2026-03-13

**Authors:** The PLOS One Staff

The word “experiences” is misspelled in the article title. The correct title is: The lived experiences of rejection sensitivity in ADHD – A qualitative exploration. The correct citation is: Rowney-Smith A, Sutton B, Quadt L, Eccles JA (2026) The lived experiences of rejection sensitivity in ADHD – A qualitative exploration. PLoS One 21(1): e0314669. https://doi.org/10.1371/journal.pone.0314669

The publisher apologizes for the error.
